# Linoleic acid metabolic pathway allows for an efficient increase of intramuscular fat content in pigs

**DOI:** 10.1186/s40104-019-0343-8

**Published:** 2019-05-07

**Authors:** Sofia Gol, Rayner González-Prendes, Lluís Bosch, Marc Tor, Josep Reixach, Ramona N. Pena, Joan Estany

**Affiliations:** 1 Department of Animal Science, Universitat de Lleida - Agrotecnio Center, 191 Rovira Roure, 25198 Lleida, Catalonia Spain; 2 Department of Chemical, Agricultural and Food Technology Engineering, Universitat de Girona, Campus de Montilivi, 17071 Girona, Catalonia Spain; 3 Selección Batallé S.A., Av. Segadors s/n, 17421 Riudarenes, Catalonia Spain

**Keywords:** Arachidonic, Genetic parameters, Intramuscular fat, Linoleic, Meat quality, Swine

## Abstract

**Background:**

Intramuscular fat (IMF) content is a relevant trait for high-quality meat products such as dry-cured ham, but increasing IMF has the undesirable correlated effect of decreasing lean growth. Thus, there is a need to find selection criteria for IMF independent from lean growth. In pigs, the proportion of linoleic (C18:2) and arachidonic (C20:4) acids decline with fat deposition and therefore they can be considered as indicators of fatness. The aim of this research was to estimate the genetic variation for C18:2 and C20:4 in IMF and their genetic correlations with IMF and lean growth traits, with the objective to assess their potential as specific biomarkers of IMF. The analysis was conducted using a full-pedigreed Duroc resource line with 91,448 records of body weight and backfat thickness (BT) at 180 days of age and 1371 records of fatty acid composition in the muscle *gluteus medius*.

**Results:**

The heritability estimates for C18:2 and C20:4 in IMF, whether expressed in absolute (mg/g of muscle) or in relative (mg/g of fatty acid) terms, as well as for their ratio (C20:4/C18:2), were high (> 0.40), revealing that the C18:2 to C20:4 pathway is subjected to substantial genetic influence. Litter effects were not negligible, with values ranging from 8% to 15% of the phenotypic variance. The genetic correlations of C18:2 and C20:4 with IMF and BT were negative (− 0.75 to − 0.66, for IMF, and − 0.64 to − 0.36, for BT), if expressed in relative values, but almost null (− 0.04 to 0.07), if expressed in absolute values, except for C18:2 with IMF, which was highly positive (0.88). The ratio of C20:4 to C18:2 also displayed a stronger genetic correlation with IMF (− 0.59) than with BT (− 0.10).

**Conclusions:**

The amount of C18:2 in muscle can be used as an IMF-specific biomarker. Selection for the absolute amount of C18:2 is expected to deliver a similar response outcome as selection for IMF at restrained BT. Further genetic analysis of the C18:2 metabolic pathway may provide new insights into differential fat deposition among adipose tissues and on candidate genes for molecular markers targeting specifically for one of them.

## Introduction

Linoleic acid (C18:2) is a major ingredient of feeds and the most abundant PUFA in pig adipose tissue and muscle [1]. Since pigs are not able to synthesize C18:2, its amount in tissue is highly correlated with dietary intake. Of all fatty acids, C18:2 shows the greatest tissue response to dietary levels [2]. In the cells, C18:2 can be either elongated to eicosadienoic acid (C20:2) or transformed into arachidonic acid (C20:4) [3]. Thus, although C20:2 and C20:4 are sourced from diet, they can also be endogenously synthesised. The proportion of C18:2 and C20:4 in pig adipose tissue and muscle declines with fat deposition [1], a phenomenon that has been explained by the relative lower concentration of dietary fatty acids in adipose cells as de novo synthesis of fatty acids progresses. For this reason, the content of C18:2 in adipose tissue content has been considered as an indicator of fatness [4].

Intramuscular fat (IMF) content and fatty acid composition are relevant traits for high-quality Mediterranean meat products such as dry-cured ham. Increasing IMF has the undesirable correlated effect of decreasing lean growth, so that, in this scenario, a common commercial target is to find selection criteria for IMF independent from lean growth [5]. Although substantial genetic variation between [6] and within [7, 8] genetic types for fatty acid composition has been reported, the potential of C18:2 and its long chain products as specific indicators of IMF has not yet been fully addressed. Thus, the aim of this study was to estimate the genetic relationships of C18:2, C20:2 and C20:4 in IMF with lean growth traits in a purebred Duroc population and then to discuss their potential use as a means to direct selection solely for IMF.

## Methods

### Animals and sample collection

Data from a purebred Duroc line (Selección Batallé, Riudarenes, Girona, Spain) were used for the analyses [9, 10]. The line was completely closed in 1991 and since then it has been selected for an index including body weight (BW), backfat thickness (BT), and IMF. The data set used for the estimation of genetic parameters consisted of 162,494 pedigree-connected pigs, from which 91,525 had at least 1 recorded trait (Table 1). At about 75 days of age pigs were moved to the fattening units, where they were allocated by sex in pens of 8 to 12 individuals and were given ad libitum access to commercial diets. Pigs were performance-tested at an average age of 177 d for BW and BT. Backfat thickness was ultrasonically measured at 5 cm off the midline between the third and fourth last ribs using the portable equipment Piglog 105 (Frontmatec, Kolding, Denmark). Since 2002, 1,371 purebred barrows used for producing dry-cured ham were sampled to record IMF content and fatty acid composition in *gluteus medius* muscle. These barrows were raised in 23 batches to slaughter at around 215 days of age. From 160 days of age they were fed a finishing diet (Esporc, Riudarenes, Girona, Spain) including around 6.0% fat (27% C18:2 and 0.3% C20:4 of total fatty acids). All barrows were slaughtered in a slaughterhouse equipped with a carbon dioxide stunning system (Butina ApS, Holbaek, Denmark). After chilling for about 24 h at 2 °C, a sample of at least 50 g of the *gluteus medius* muscle was taken from the left side ham. Muscle samples were immediately vacuum packaged and stored at − 20 °C until required for IMF and fatty acid determinations. The number of records, sires, dams, and litters used for each analyzed trait is given in Table 1.

**Table 1:** Description of the data set used in the analyses

Items	No. of pigs	No. of sires	No. of dams	No. of litters	Mean	Standard deviation
Pedigree	162,494	1032	32,767	38,253	–	–
Trait^a^						
Body weight at test, kg	91,448	670	22,297	38,226	105.4	12.4
Backfat thickness at test, mm	91,061	670	22,256	38,109	16.1	3.4
Intramuscular fat, %	1371	179	752	755	4.9	1.9
C18:2, mg/g FA	1371	179	752	755	113.4	21.9
C20:2, mg/g FA	1371	179	752	755	5.4	1.2
C20:4, mg/g FA	1371	179	752	755	13.5	6.3
C18:2, mg/g	1371	179	752	755	17.1	4.2
C20:2, mg/g	1371	179	752	755	0.8	0.3
C20:4, mg/g	1371	179	752	755	1.9	0.7
C20:2 / C18:2 (×100)	1371	179	752	755	4.8	0.8
C20:4 / C18:2 (× 100)	1371	179	752	755	11.9	4.8
Covariates						
Age at test, d	91,448	670	22,297	38,226	177.2	10.6
Age at slaughter, d	1371	179	752	755	213.5	9.9

^a^Intramuscular fat content and fatty acid composition in the muscle *gluteus medius*. Linoleic acid (C18:2), eicosadienoic acid (C20:2) and arachidonic acid (C20:4) are expressed in relative (mg/g of fatty acid (FA)) or in absolute value (mg/g of dry muscle)

### Fatty acid analysis

Defrosted muscle samples were freeze-dried and pulverized prior to fat analysis. Intramuscular fat content and fatty acid composition was determined in duplicate by quantitative determination of the individual fatty acids by gas chromatography [11]. Total fatty acid methyl esters from both neutral lipids and phospholipids were directly obtained by transesterification using a solution of 20% boron trifluoride in methanol [12]. Methyl esters were determined by gas chromatography using a capillary column SP2330 (30 m × 0.25 mm; Supelco, Bellefonte, PA) and a flame ionization detector with helium as carrier gas. Runs were made with a constant column-head pressure of 172 kPa. The oven temperature program increased from 150 to 225 °C at 7 °C/min and injector and detector temperatures were both 250 °C. The quantification was carried out through area normalization with an external mixture of fatty acid methyl esters (Supelco 37 Component FAME Mix. Sigma, Tres Cantos, Madrid) after adding into each sample 1,2,3-tripentadecanoylglycerol as internal standard. Fatty acids were identified by comparing their relative retention times with those of the external standard and confirmed by mass spectrometry. The amount of C18:2, C20:2 and C20:4 was expressed either in absolute (mg/g of dry muscle) or in relative (mg/g of total fatty acids) values. The total amount of fatty acids was calculated as the sum of C14:0, C16:0, C16:1*n-9*, C18:0, C18:1*n-7*, C18:1*n-9*, C18:2*n-6*, C18:3*n-3*, C20:0; C20:1*n-7*, C20:2*n-6* and C20:4*n-6*. Intramuscular fat content was calculated as the sum of each individual fatty acid expressed as triglyceride equivalents [13] on a wet tissue basis. Means and standard deviations of the investigated fatty acids and their associated ratios (C20:2/C18:2 and C20:4/C18:2) are shown in Table 1.

### Genetic parameters

Genetic parameters for BW, BT, IMF, C18:2, C20:2 and C20:4 (fatty acids alternatively expressed in relative or absolute values) were estimated fitting a 6-trait multivariate animal model. In matrix notation, the model used was **y**_*i*_ = **X**_*i*_ **b**_*i*_ + **Z**_*i*_ **a**_***i***_ + **W**_*i*_ **c**_*i*_ + **e**_*i*_, where **y**_*i*_ is the vector of observations for trait *i* (BW, BT, IMF, C18:2, C20:2 and C20:4); **b**_*i*_, **a**_*i*_, **c**_*i*_, and **e**_*i*_ are the vectors of systematic, additive genetic, litter, and residual effects, respectively; and **X**_*i*_, **Z**_*i*_, and **W**_*i*_, are the known incidence matrices that relate **b**_*i*_, **a**_*i*_, and **c**_*i*_ with **y**_*i*_, respectively. Systematic effects for BW and BT were the batch (1,032 levels), gender (3 levels; males, females, and barrows), and age at measurement as a covariate. Pigs tested at the same time and in the same farm unit were considered as one batch. The same model was used for IMF and fatty acid composition but with systematic effects only including the batch at slaughter (23 levels) and the age at slaughter as a covariate.

Genetic parameters were estimated in a Bayesian setting, in line with the methodology described in Ros-Freixedes et al. [10], and using Gibbs sampling with the TM software [14]. The traits were assumed to be conditionally normally distributed as [**y**_*i*_ | **b**_*i*_ **a**_*i*_ **c**_*i*_ **R]** ~ N (**Xb**_*i*_ + **Za**_*i*_ + **Wc**_*i*_, **R**), where **R** was the (co) variance matrix. Sorting records by trait, and pig within trait, **R** could be written as **R**_0_ ⊗ **I**, with **R**_0_ being the 6 × 6 residual (co) variance matrix between the six traits analyzed and **I** an identity matrix of appropriate order. Flat priors were used for **b**_*i*_ and residual (co) variance components. Additive genetic and litter values, conditionally on the associated (co) variance components, were both assumed to be multivariate normally distributed with mean zero and with (co) variance **G** ⊗ **A** and **C** ⊗ **I**, respectively, where **A** was the numerator relationship matrix, **G** was the 6 × 6 genetic relationship matrix between the six traits, and **C** was the 6 × 6 (co) variance matrix between litter effects. The matrix **A** was calculated using all the pedigree information summarised in Table 1. Flat priors were used for additive and litter (co) variance components. Statistical inferences (means, standard deviations and HPD95) were derived from the samples of the marginal posterior distribution using a unique chain of 1,000,000 iterations, where the first 200,000 were discarded and one sample out of 100 iterations retained. Statistics of marginal posterior distributions and the convergence diagnostics were obtained using the BOA package [15]. Convergence was tested using the *Z*-criterion of Geweke and visual inspection of convergence plots. The genetic parameters for C20:2/C18:2 and C20:4/C18:2 were estimated separately using the same procedure but with a 4-trait multivariate animal model including BW, BT and IMF.

### Expected responses

Expected genetic responses in BW, BT and IMF were predicted after selecting for IMF, BT, IMF at constant BT and C18:2. A population with discrete generations was simulated in which 40 boars were randomly mated to 400 sows with a mating ratio of 1 boar to 10 sows. The breeding scheme consisted of one selection stage resulting in the top 25% males and 50% females. It was assumed that two males and two females from the offspring of each sow were performance-tested for BW and BT and three paternal half-sibs of different dams were used for IMF and C18:2 determinations. Pigs were assumed to be selected for one trait at a time, but using the records taken only on the traits included in the selection criterion or in all traits. Selection response was predicted by deterministic simulation of a one-stage selection scheme with discrete generations using the program SelAction [16]. The program accounts for reduction in variance due to selection [17] and corrects selection intensities for finite population size and for the correlation between index values of family members [18].

## Results

The content of C18:2, C20:2 and C20:4 in IMF is influenced by the pig’s genetic background (Table 2). The estimates of the heritability for C18:2, C20:2 and C20:4, expressed in relative terms, were high, with values ranging from 0.43 (C20:2) to 0.72 (C18:2). Moreover, the proportion of the phenotypic variance due to litter effects was not negligible for these fatty acids, showing values around 0.10 (0.08, for C18:2 and C20:2, and 0.13, for C20:4, with a probability of 95% of being greater than 0.04, 0.03 and 0.08, respectively). The genetic correlations among them were all positive, high for those involving C18:2 (> 0.60), and low for that between C20:2 and C20:4 (0.19; HPD95 [− 0.07, 0.49]). Litter correlations were in line with genetic correlations. If C18:2, C20:2 and C20:4 were expressed in absolute values, the heritabilities and litter variances displayed a similar pattern as for relative values. Thus, the heritabilities ranged from 0.42 (C20:4) to 0.61 (C20:2) and litter variances stayed around 10% of the phenotypic variance. The genetic correlation structure among them, however, differed when expressed in absolute terms. All of the genetic correlations were still positive, but only that between C18:2 and C20:2 remained high (0.96; HPD95 [0.94, 0.98]). The genetic correlations of C20:4, in absolute value, with C18:2 (0.15; HPD95 [− 0.11, 0.41]) and C20:2 (0.12; HPD95 [− 0.09, 0.48]) did not exceed 0.15.

**Table 2:** Posterior means (standard deviation) of heritability (bold diagonal), genetic correlations (above diagonal), litter correlations (under diagonal), litter variance in proportion to the phenotypic variance (l^2^), additive genetic variance (σ^2^_a_), litter variance (σ^2^_l_), and residual variance (σ^2^_e_) for linoleic acid (C18:2), eicosadienoic acid (C20:2) and arachidonic acid (C20:4), expressed in either relative (mg/g of fatty acid) or absolute value (mg/g of dry muscle)

Items	Trait		
	C18:2	C20:2	C20:4
Relative value			
C18:2	**0.72 (0.09)**	0.71 (0.06)	0.61 (0.10)
C20:2	0.74 (0.12)	**0.43 (0.08)**	0.19 (0.15)
C20:4	0.41 (0.17)	0.04 (0.21)	**0.53 (0.08)**
l^2^	0.08 (0.03)	0.08 (0.02)	0.13 (0.03)
σ^2^_a_	223.89 (35.14)	0.31 (0.07)	13.83 (2.43)
σ^2^_l_	25.93 (9.50)	0.06 (0.02)	3.42 (0.87)
σ^2^_e_	60.00 (20.48)	0.36 (0.04)	8.89 (1.82)
Absolute value			
C18:2	**0.58 (0.09)**	0.96 (0.01)	0.15 (0.15)
C20:2	0.95 (0.02)	**0.61 (0.08)**	0.12 (0.14)
C20:4	−0.02 (0.19)	−0.08 (0.19)	**0.42 (0.10)**
l^2^	0.08 (0.02)	0.08 (0.02)	0.15 (0.03)
σ^2^_a_	9.41 (1.85)	0.04 (0.01)	0.10 (0.03)
σ^2^_l_	1.31 (0.39)	0.01 (0.00)	0.04 (0.01)

The estimates of the genetic parameters for BW, BT and IMF showed little differences if fatty acids in the multivariate model were expressed in relative or absolute value. Because of this, only the estimates for absolute values are given (Table 3). As expected, the estimates were in close agreement with previous results in this Duroc line [5]. The genetic correlation between IMF and BT was positive but moderate (0.32). The genetic correlations of C18:2, C20:2 and C20:4, in relative value, with BW, BT and IMF are shown in Table 4. All of them were negative, ranging from − 0.75 (HPD95 [− 0.87, − 0.60]), for C20:4 and IMF, to − 0.17 (HPD95 [− 0.46, 0.13]), for C20:2 and IMF. Interestingly, the genetic correlation of C20:4 with IMF was lower than with BT (− 0.36; HPD95 [− 0.56, − 0.16]), while the opposite situation happened for C20:2, where the genetic correlation with IMF was greater than with BT (− 0.48; HPD95 [− 0.65, − 0.28]). This was not the case for C18:2, which presented similar genetic correlations with IMF (− 0.66; HPD95 [− 0.84, − 0.49]) and BT (− 0.64; HPD95 [− 0.77, − 0.51]). A different genetic correlation structure emerged when fatty acids were expressed in absolute value (Table 5). In this case, all genetic correlations were very low (from − 0.04, HPD95 [− 0.26, 0.18], to 0.15, HPD95 [− 0.02, 0.34]), except those of C18:2 and C20:2 with IMF, which were very high (> 0.88). This result indicates that C18:2 and C20:2 in IMF, expressed in absolute terms, are highly specific to IMF.

**Table 3:** Posterior mean (standard deviation) of heritability (bold diagonal), genetic correlations (above diagonal), litter correlations (under diagonal), litter variance in proportion to the phenotypic variance (l^2^), additive genetic variance (σ^2^_a_), litter variance (σ^2^_l_), and residual variance (σ^2^_e_) for body weight (BW), backfat thickness (BT) and intramuscular fat content (IMF)

Items	Trait		
	BW	BT	IMF
BW	**0.39 (0.01)**	0.65 (0.01)	0.22 (0.10)
BT	0.61 (0.02)	**0.53 (0.01)**	0.32 (0.08)
IMF	−0.28 (0.26)	0.27 (0.23)	**0.57 (0.10)**
l^2^	0.09 (0.00)	0.06 (0.00)	0.08 (0.03)
σ^2^_a_	37.27 (1.30)	5.28 (0.14)	1.89 (0.43)
σ^2^_l_	8.61 (0.34)	0.55 (0.03)	0.25 (0.10)
σ^2^_e_	49.10 (0.74)	4.08 (0.08)	1.12 (0.27)

**Table 4:** Posterior mean (standard deviation) of the genetic correlation, litter correlation and residual correlation of linoleic acid (C18:2), eicosadienoic acid (C20:2) and arachidonic acid (C20:4), expressed in relative value (mg/g of fatty acid), with body weight (BW), backfat thickness (BT) and intramuscular fat content (IMF)

Items	Trait		
	BW	BT	IMF
Genetic correlation			
C18:2	−0.39 (0.08)	−0.64 (0.07)	− 0.66 (0.10)
C20:2	−0.29 (0.11)	− 0.48 (0.09)	− 0.17 (0.15)
C20:4	− 0.24 (0.12)	− 0.36 (0.10)	− 0.75 (0.07)
Litter correlation			
C18:2	0.20 (0.18)	−0.20 (0.20)	− 0.53 (0.21)
C20:2	−0.19 (0.17)	−0.31 (0.24)	− 0.10 (0.30)
C20:4	0.33 (0.18)	−0.16 (0.18)	−0.58 (0.15)
Residual correlation			
C18:2	−0.16 (0.09)	−0.20 (0.09)	− 0.50 (0.20)
C20:2	−0.13 (0.06)	− 0.15 (0.06)	−0.11 (0.12)
C20:4	−0.13 (0.08)	−0.22 (0.08)	− 0.43 (0.11)

**Table 5:** Posterior mean (standard deviation) of the genetic correlation, litter correlation and residual correlation of linoleic acid (C18:2), eicosadienoic acid (C20:2) and arachidonic acid (C20:4), expressed in absolute value (mg/g of dry muscle), with body weight (BW), backfat thickness (BT) and intramuscular fat content (IMF)

Items	Trait		
	BW	BT	IMF
Genetic correlation			
C18:2	0.07 (0.11)	0.07 (0.10)	0.88 (0.03)
C20:2	0.10 (0.11)	0.15 (0.10)	0.91 (0.03)
C20:4	0.00 (0.15)	−0.04 (0.11)	0.06 (0.13)
Litter correlation			
C18:2	−0.13 (0.24)	0.21 (0.26)	0.85 (0.07)
C20:2	−0.28 (0.26)	0.14 (0.27)	0.84 (0.08)
C20:4	0.01 (0.20)	−0.11 (0.21)	−0.03 (0.20)
Residual correlation			
C18:2	−0.03 (0.08)	−0.05 (0.10)	0.69 (0.12)
C20:2	−0.03 (0.09)	−0.04 (0.11)	0.65 (0.13)
C20:4	−0.12 (0.08)	−0.21 (0.07)	0.17 (0.12)

The estimates of the genetic parameters for the ratios related to the transformation efficiency of C18:2 into C20:2 and C20:4 confirmed that the linoleic to arachidonic acid pathway is subjected to genetic determinism (Table 6). Both ratios showed a high heritability, in the range of 0.40 to 0.50, and relevant litter effects, particularly for C20:4/C18:2, where they explained 15% of the phenotypic variance. Similarly to C20:2 and C20:4, the genetic correlation of C20:4/C18:2 with IMF (− 0.59; HPD95 [− 0.82, − 0.33]) was stronger than with BT (− 0.10; HPD95 [− 0.32, 0.10]), while that of C20:2/C18:2 with IMF (0.76; HPD95 [0.61, 0.87]) was greater than with BT (0.36; HPD95 [0.15, 0.54]). Taken together, this correlation pattern corroborates the potential of the C18:2 metabolic pathway as a candidate route to hold molecular markers specifically targeting IMF.

**Table 6:** Posterior mean (standard deviation) of additive genetic variance (σ^2^_a_), litter variance (σ^2^_l_), and residual variance (σ^2^_e_), heritability (h^2^) and litter variance in proportion to the phenotypic variance (l^2^) for the eicosadienoic acid (C20:2) to linoleic acid (C18:2) ratio (C20:2/C18:2) and the arachidonic acid (C20:4) to C18:2 ratio (C20:4/C18:2), and their genetic, litter and residual correlations with body weight (BW), backfat thickness (BT) and intramuscular fat content (IMF)

Items	Trait (×100)	
	C20:2/C18:2	C20:4/C18:2
σ^2^_a_	0.18 (0.03)	5.83 (1.48)
σ^2^_l_	0.02 (0.01)	1.72 (0.50)
σ^2^_e_	0.25 (0.02)	4.14 (0.84)
h^2^	0.40 (0.06)	0.50 (0.11)
l^2^	0.05 (0.02)	0.15 (0.04)
Genetic correlation		
BW	0.24 (0.12)	−0.09 (0.14)
BT	0.36 (0.10)	−0.10 (0.12)
IMF	0.76 (0.07)	−0.59 (0.12)
Litter correlation		
BW	−0.38 (0.24)	0.18 (0.19)
BT	−0.04 (0.28)	−0.19 (0.20)
IMF	0.64 (0.19)	−0.52 (0.19)
Residual correlation		
BW	−0.03 (0.06)	−0.10 (0.09)
BT	0.01 (0.06)	−0.16 (0.09)
IMF	0.27 (0.08)	−0.43 (0.10)

To illustrate and explore the potential of using C18:2 as a selection criterion for IMF, the expected genetic response on a basic breeding scheme was predicted using different selection criteria and data availability scenarios (Table 7). With the genetic parameters estimated here, it is shown that, in terms of expected response, selection for absolute values of C18:2 parallels selection for IMF at restrained BT. Although both criteria rendered similar results for IMF (from 80% to 92% of the direct response), selection for C18:2 led to higher responses in both BW (32–35% vs. 6% of the direct response) and BT (21–24% of the direct response vs. no change). Thus, pigs selected for C18:2 (in absolute value) are expected to show at least the same lean growth and IMF than pigs selected for IMF at restrained BT. Results anticipate that the detrimental effect of selection for C18:2 on carcass lean content should be offset by the increase in body weight.

**Table 7:** Expected response per generation in body weight (BW), backfat thickness (BT) and intramuscular fat (IMF) to selection for BW, BT, IMF at restricted BT (ΔBT = 0) and C18:2 (mg/g of dry muscle) on a basic pig breeding scheme when records used for selection were taken only on the selected traits or on all traits ^a^

Selection criterion	Expected response		
	BW, kg	BT, mm	IMF, %
Records on selected traits ^b^			
IMF	100	100	100
BT	644	681	70
IMF at ΔBT = 0	6	0	92
C18:2 mg/g	32	21	87
Records on all traits ^c^			
IMF	100	100	100
BT	156	165	61
IMF at ΔBT = 0	6	0	80
C18:2 mg/g	35	24	83

^a^The breeding scheme consisted of one selection stage resulting in the top 25% males and 50% females of the offspring of 40 boars and 400 sows (ratio of 1 boar to 10 sows). Two males and two females from the offspring of each sow were performance-tested for BW and BT and three paternal half-sibs of different dams were used for IMF and C18:2 determinations. Pigs were selected for one trait at a time, but using the records taken only on the traits included in the selection criterion or in all traits

^b^Responses in percentage relative to responses to selection for IMF (0.39 kg, 0.21 mm and 0.40%, for BW, BT and IMF, respectively), which are set to 100

^c^Responses in percentage relative to responses to selection for IMF (1.51 kg, 0.88 mm and 0.46%, for BW, BT and IMF, respectively), which are set to 100

## Discussion

**Fig. 1:**
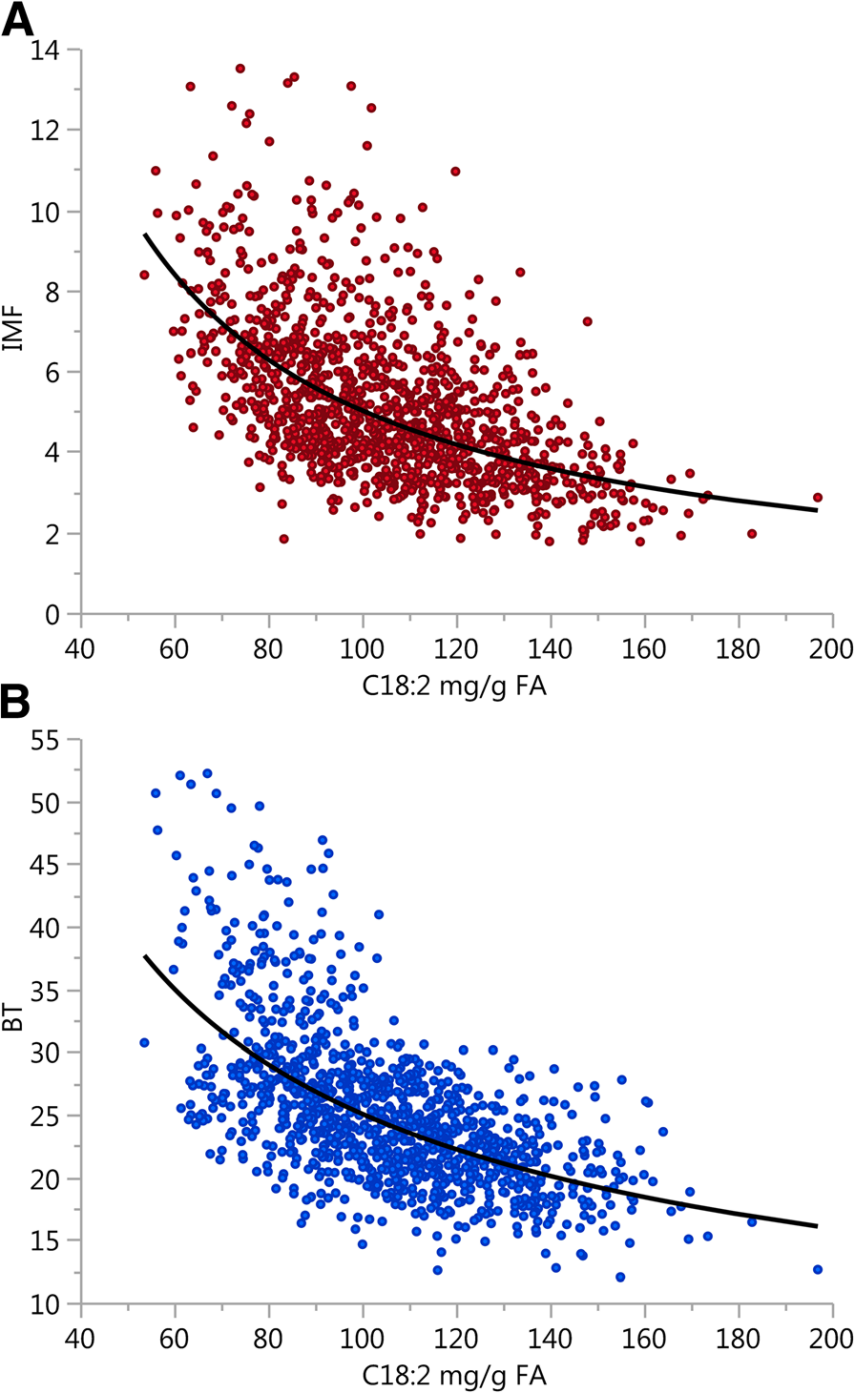
Relationship between the relative amount of linoleic acid in muscle and (**a**) intramuscular fat or (**b**) subcutaneous backfat thickness. The linoleic acid (C18:2, in mg/g of fatty acid (FA)) is negatively related to both intramuscular fat content (IMF, %; log (IMF) = 6.26–1.00 × log (C18:2); *R*^2^: 0.36) and backfat thickness (BT, mm; log (BT) = 6.24–0.65 × log (C18:2); *R*^2^: 0.39)

The C18:2 present in the adipose tissue of pigs derives from the diet, as mammals cannot synthetize this fatty acid. Linoleic acid is a major fat ingredient of commercial pig diets, mostly composed of grains and oils very rich in C18:2. Hence, C18:2 is relatively abundant in pigs, particularly as compared to ruminant species [4]. Relative C18:2 percentages in muscle, depending on the breed, diet and muscle, vary from 5% to 20% of the total fatty acids [6]. Values found in our experiment, of around 11%, fall within the average. Once in the tissue, C18:2 can be transformed to C20:4 by two metabolic routes. On one hand, C18:2 can be desaturated to γ-linolenic acid, and then successively elongated and desaturated to C20:4. On the other hand, C18:2 can be elongated to C20:2 and then desaturated twice to C20:4. As compared to reported values (2–12%; [6]), we observed a relatively high C20:4 to C18:2 ratio (11.9%). This would point to a relatively active endogenous transformation of ingested fatty acids, since dietary C20:4/C18:2 was only about 1%. As a result of the *de novo* fatty acid biosynthesis, the relative amount of C18:2 declines, as also happened here (Fig. 1). This has led to propose C18:2 as a candidate biomarker of both feed intake [19] and fatness [4]. But we can go a step further and hypothesize that, because the rate and timing of fat deposition differs between adipose tissues, with IMF developing later than subcutaneous fat [20], C18:2 in IMF, and by extension the other fatty acids involved in its metabolism, could be IMF-specific enough to capture that part of the variability of IMF which is independent of BT (Fig. 1). Previous results in this Duroc line have evidenced that the correlation pattern of fatty acid composition among different muscles and with subcutaneous fat is far from unity [21].

To examine this hypothesis we first have shown that C18:2 and C20:4 displayed genetic variability, whether expressed in relative or absolute values. In general, our estimates of the heritability for C18:2 and C20:4 were higher than others published so far, which ranged, for C18:2, from 0.24 to 0.55 [8, 22–26] and, for C20:4, from 0.15 to 0.56 [24–26]. The estimate of the heritability for C20:2 was also relatively high and in line with the only one published so far [26]. Most of the reported estimates, however, were adjusted either for carcass weight, IMF or total fatty acids, which may affect the estimates downwards [26]. Interestingly, we have also found that C18:2, C20:2 and particularly C20:4 display a relevant litter effect. A similar effect has been reported by Ibáñez-Escriche et al. [27] in Iberian in pigs. Variation across litters for C18:2 and C20:4 can arise from maternal effects due to differential nutrient intake. Maternal nutrition has been seen to influence fetal programming [28] and milk yield and composition [29], which are known to influence adipogenesis and therefore meat fatty acid composition. Altogether, these findings evidenced that the linoleic to arachidonic acid pathway has a strong genetic background and is not unresponsive to common environmental litter effects, which, as shown, can remain for a long time after weaning.

**Fig. 2:**
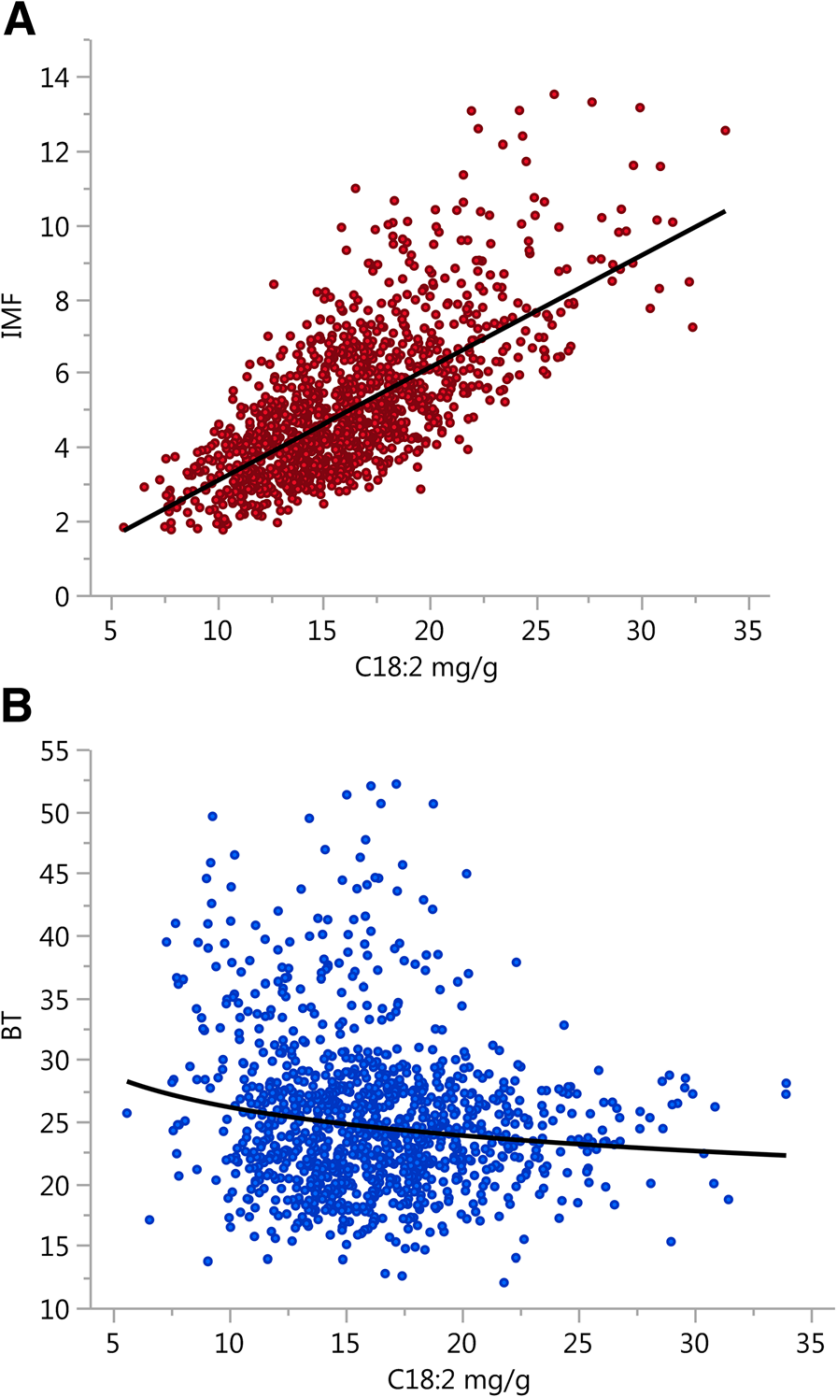
Relationship between the absolute amount of linoleic acid in muscle and (**a**) intramuscular fat or (**b**) subcutaneous backfat thickness. The linoleic acid (C18:2, in mg/g of dry muscle) is positively related to intramuscular fat content (IMF, %; log (IMF) = −1.17 + 0.99 × log (C18:2); *R*^2^:0.50) but not to backfat thickness (BT, mm)

Secondly, the genetic correlation structure of C18:2, C20:2 and C20:4 with IMF and BT showed that these fatty acids have potential to be used as IMF- or BT-specific biomarkers, although this depends critically on how they are expressed. Thus, in line with the results in Suzuki et al. [23], if expressed in relative value, all three fatty acids were negatively correlated with IMF and BT, whereas, if expressed in absolute value, only C18:2 and C20:2 were correlated with IMF, and positively. This discrepancy makes the absolute amount of C18:2 and C20:2 in IMF a criterion of choice for discriminating IMF against BT. Of these two fatty acids, C18:2 is a more feasible biomarker given its abundance, which makes determinations less sensitive to measurement errors. This dual relationship of C18:2 with IMF (positive) and BT (null) can be directly viewed upon depicting the raw phenotypes of IMF and BT against the absolute amount of C18:2 in IMF (Fig. 2). We were unable to find in the literature other estimates of genetic parameters for fatty acids in absolute value. Efficiency ratios did not improve the potential of C18:2 for specific targeting of IMF. Although both C20:2/C18:2 and C20:4/C18:2 were also more linked to IMF than BT, their correlation structure with IMF and BT was less uneven than in C18:2.

Improving IMF without compromising lean growth is a common goal in pig lines for niche and quality markets where IMF is a valued feature. In practice, this is basically done by selecting for BW and IMF and against BT, but imposing some restrictions on either IMF or BT [30]. However, since IMF and BT are positively correlated, undesirable changes in BT to selection for IMF and vice versa can easily happen. For this reason, there have been attempts to find indirect selection criteria, such as circulating lipid indicators [31, 32], targeting specifically to one of them. The favourable genetic correlation pattern of C18:2 (in absolute value) with IMF and BT calls for exploring C18:2 as one of such criteria. Expected responses in IMF, BT and BW indicate that selecting for the absolute amount of C18:2 is at least as efficient to selecting for IMF at restrained BT. In other words, the absolute amount of C18:2 in IMF is able to capture most of the variance of IMF that is independent of BT and, in this way, it behaves as an IMF-specific biomarker. Nonetheless, the use of C18:2 as an indirect selection criterion for IMF presents several limitations. As happens for IMF, the most immediate is to have a feasible routine recording scheme. In this regard, the near-infrared spectroscopy (NIRS) allows continuous non-invasive phenotyping of meat quality traits at a fair cost. This technology has been already used to determine the fatty acid composition of the subcutaneous fat in Iberian [22] as well as in Duroc and in Landrace pigs [8]. Furthermore, new portable NIRS-based equipment is becoming available to facilitate on-line recording at the abattoir. In this scenario, C18:2 can be interpreted as an endophenotype whose variants are indirectly captured by NIRS spectra [33].

We have used the *gluteus medius* as the muscle of choice, as it is a representative muscle of the ham, the most valuable entire piece for the dry-cured meat product industry. Other reference muscles could have been used for this purpose, such as muscle *longissimus thoracis*. Although results may differ among them, at least for these two muscles the differences are not expected to be substantial [21]. This has been confirmed using a subset of pigs having also data from *longissimus thoracis.* In this muscle, the genetic correlation pattern of C18:2 (in absolute value) with IMF (0.82; HPD95 [0.69, 0.88]) and BT (0.23; HPD95 [0.06, 0.41]) was in line with the observed in gluteus medius. Alternatively, the fatty acid composition in subcutaneous fat could also work as a BT-specific biomarker. Current evidence indicates that the C18:2 content in subcutaneous fat is negatively correlated to BT [23] and uncorrelated to IMF [22]. If confirmed, this can be an option for pig lines already performing at an optimum level of IMF, where selection is focused on lean growth at restrained IMF. The use of C18:2 as selection criterion may draw the idea that fat will become more polyunsaturated and with less oleic acid, thereby affecting adversely key attributes of dry-cured products. However, the exact opposite occurs. Estimates obtained in this Duroc population indicate that the absolute value of C18:2 is genetically positively correlated with the oleic acid content, regardless of how it is expressed, either in absolute (0.77; HPD95 [0.67, 0.86]) or in relative (0.15; HPD95 [− 0.10, 0.41]) value. This provides evidence that selection for C18:2 in absolute value would not entail unfavorable correlated effects on fatty acid composition.

Over the last decades molecular markers have also raised interest as a tool to improve genetic analysis and selection. Several markers have been described to be associated with IMF, BT and fatty acid composition, although only one of them has proved to be IMF-specific [34]. The distinct association of C18:2 and C20:4 with IMF and BT described here supports the search for molecular markers in genes encoding enzymes and transcription factors involved in the C18:2 metabolic pathway. One of them is the fatty acid desaturase-2 gene (*FADS2*), a rate-limiting enzyme in the conversion of C18:2 into C20:4. The activity of FADS2 can be indirectly measured by C20:4/C18:2 and C20:2/C18:2. These two ratios are expected to decrease and increase, respectively, with IMF rather than with BT, thereby suggesting that *FADS2* could be a candidate gene to explore IMF-specific molecular markers. In this context, Gol et al. [35] found a polymorphism in the promoter region of the *FADS2* gene that modifies C20:4/C18:2 and C20:2/C18:2. The correlated effects on IMF and BT were in line with the expected, i.e., the allele showing a positive effect on C20:4/C18:2 had less absolute C18:2 and IMF, while it did not alter BT. All in all, the results obtained would confirm that quantitative biological analysis is a good approach to find new traits and candidate markers for an efficient selection for IMF and lean growth.

## Conclusions

In conclusion, our work demonstrates that the C18:2 to C20:4 pathway is subjected to genetic variation. Also, we show that the genetic (co) variation structure of the fatty acids in this pathway with IMF and BT differs by fatty acid and on whether they are expressed in absolute (mg/g of muscle) or relative values (mg/g of fatty acid). In particular, the distinct genetic relationship of C18:2 and C20:2 (in absolute values) in IMF with IMF (positive) and BT (almost null) allow us to propose them as candidate IMF-specific biomarkers. In addition, we have proved that selection for the absolute amount of C18:2 in IMF is expected to deliver a similar genetic response outcome that selection for IMF at restrained BT. The quantitative genetic analysis of the C18:2 metabolic pathway has provided new insight into the relationship between IMF and lean growth, pointing to relevant candidate genes to search for potential IMF-specific markers.

## Abbreviations

BT: Backfat thickness
BW: Body weight
C18:2: Linoleic acid
C20:2: Eicosadienoic acid
C20:4: Arachidonic acid
FADS2: Fatty acid desaturase-2
HPD95: Highest posterior density interval at 95% of probability
IMF: Intramuscular fat
NIRS: Near-infrared spectroscopy

## References

[1] Ros-Freixedes R, Estany J (2014). On the compositional analysis of fatty acids in pork. J Agric Biol Environ Stat.

[2] Nguyen LQ, Nuijens MCGA, Everts H, Salden N, Beynen AC (2003). Mathematical relationships between the intake of n-6 and n-3 polyunsaturated fatty acids and their contents in adipose tissue of growing pigs. Meat Sci.

[3] Nakamura MT, Nara TY (2004). Structure, function, and dietary regulation of Δ6, Δ5, and Δ9 desaturases. Annu Rev Nutr.

[4] Wood JD, Enser M, Fisher AV, Nute GR, Sheard PR, Richardson RI (2008). Fat deposition, fatty acid composition and meat quality: a review. Meat Sci.

[5] Estany J, Ros-Freixedes R, Tor M, Pena RN (2017). Triennial growth and development symposium: genetics and breeding for intramuscular fat and oleic acid content in pigs. J Anim Sci.

[6] Wood JD, Nute GR, Richardson RI, Whittington FM, Southwood O, Plastow G (2004). Effects of breed, diet and muscle on fat deposition and eating quality in pigs. Meat Sci.

[7] Ntawubizi M, Raes K, Buys N, De Smet S (2009). Effect of sire and sex on the intramuscular fatty acid profile and indices for enzyme activities in pigs. Livest Sci.

[8] Gjerlaug-Enger E, Kongsro J, Aass L, Ødegård J, Vangen O (2011). Prediction of fat quality in pig carcasses by near-infrared spectroscopy. Animal..

[9] Solanes FX, Reixach J, Tor M, Tibau J, Estany J (2009). Genetic correlations and expected response for intramuscular fat content in a Duroc pig line. Livest Sci.

[10] Ros-Freixedes R, Reixach J, Tor M, Estany J (2012). Expected genetic response for oleic acid content in pork. J Anim Sci.

[11] Bosch L, Tor M, Reixach J, Estany J (2009). Estimating intramuscular fat content and fatty acid composition in live and post-mortem samples in pigs. Meat Sci.

[12] Rule DC (1997). Direct transesterification of total fatty acids of adipose tissue, and of freeze-dried muscle and liver with borontrifluoride in methanol. Meat Sci.

[13] AOAC Official Method 996.06: Fat (total, saturated, and monounsaturated) in foods hydrolytic extraction gas chromatographic method. Official methods of analysis. 1997;16th ed. 18 Suppl.

[14] Legarra A, Varona L, López de Maturana E. TM: Threshold model.2011. http://snp.toulouse.inra.fr/~alegarra/manualtm.pdf. Accessed 19 Mar 2018.

[15] Smith BJ. Bayesian output analysis program (BOA), version 1.1.5. The University of Iowa. 2005. http://www.public-health.uiowa.edu/boa. Accessed 3 Aug 2018.

[16] Rutten MJM, Bijma P, Woolliams JA, Van Arendonk JAM (2002). SelAction: software to predict selection response and rate of inbreeding in livestock breeding programs. J Hered.

[17] Bulmer MG (1971). The effect of selection on genetic variability. Am Nat.

[18] Meuwissen THE (1991). Reduction of selection differentials in finite populations with a nested full-half sib family structure. Int Biom Soci.

[19] Baylin A, Campos H. The use of fatty acid biomarkers to reflect dietary intake. Curr Opin Lipidol. 2006;(1):22–7. 10.1097/01.mol.0000199814.46720.83 16407712

[20] Du M, Huang Y, Das A. Manipulating mesenchymal progenitor cell differentiation to optimize performance and carcass value of beef cattle. J Anim Sci. 2013:1419–27. 10.2527/jas.2012-5670 23100595

[21] Ros-Freixedes R, Reixach J, Bosch L, Tor M, Estany J (2014). Genetic correlations of intramuscular fat content and fatty acid composition among muscles and with subcutaneous fat in Duroc pigs. J Anim Sci.

[22] Fernández A, De Pedro E, Núñez N, Silió L, García-Casco J, Rodríguez C (2003). Genetic parameters for meat and fat quality and carcass composition traits in Iberian pigs. Meat Sci.

[23] Suzuki K, Ishida M, Kadowaki H, Shibata T, Uchida H, Nishida A (2006). Genetic correlations among fatty acid compositions in different sites of fat tissues, meat production, and meat quality traits in Duroc pigs. J Anim Sci.

[24] Sellier P, Maignel L, Bidanel JP (2010). Genetic parameters for tissue and fatty acid composition of backfat, perirenal fat and longissimus muscle in large white and landrace pigs. Animal..

[25] Casellas J, Noguera JL, Reixach J, Díaz I, Amills M, Quintanilla R (2010). Bayes factor analyses of heritability for serum and muscle lipid traits in Duroc pigs. J Anim Sci.

[26] Ntawubizi M, Colman E, Janssens S, Raes K, Buys N, de Smet S (2010). Genetic parameters for intramuscular fatty acid composition and metabolism in pigs. J Anim Sci.

[27] Ibáñez-Escriche N, Magallón E, Gonzalez E, Tejeda JF, Noguera JL (2016). Genetic parameters and crossbreeding effects of fat deposition and fatty acid profiles in Iberian pig lines. J Anim Sci.

[28] Du M, Wang B, Fu X, Yang Q, Zhu MJ (2015). Fetal programming in meat production. Meat Sci.

[29] Jin C, Fang Z, Lin Y, Che L, Wu C, Xu S (2017). Influence of dietary fat source on sow and litter performance, colostrum and milk fatty acid profile in late gestation and lactation. J Anim Sci.

[30] Ros-Freixedes R, Reixach J, Bosch L, Tor M, Estany J (2013). Response to selection for decreased backfat thickness at restrained intramuscular fat content in Duroc pigs. J Anim Sci.

[31] Estany J, Tor M, Villalba D, Bosch L, Gallardo D, Jiménez N (2007). Association of CA repeat polymorphism at intron 1 of insulin-like growth factor (IGF-I) gene with circulating IGF-I concentration, growth, and fatness in swine. Physiol Genomics.

[32] Muñoz R, Tor M, Estany J (2012). Relationship between blood lipid indicators and fat content and composition in Duroc pigs. Livest Sci.

[33] Rincent R, Charpentier J-P, Faivre-Rampant P, Paux E, Le Gouis J, Bastien C, et al. Phenomic selection is a low-cost and high-throughput method based on indirect predictions: proof of concept on wheat and poplar.G3. 2018;1–15. 10.1534/g3.118.200760 PMC6288839 30373914

[34] Pena RN, Ros-Freixedes R, Tor M, Estany J. Genetic marker discovery in complex traits: a field example on fat content and composition in pigs. Int J Mol Sci. 2016. 10.3390/ijms17122100. 10.3390/ijms17122100 PMC5187900 27983643

[35] Gol S, Pena RN, Rothschild MF, Tor M, Estany J (2018). A polymorphism in the fatty acid desaturase-2 gene is associated with the arachidonic acid metabolism in pigs. Sci Rep.

